# Hepatitis C in Haematological Patients

**DOI:** 10.1155/2010/961359

**Published:** 2010-08-25

**Authors:** Y. Y. Hwang, R. H. S. Liang

**Affiliations:** Division of Haematology, Department of Medicine, Queen Mary Hospital, The University of Hong Kong, Pokfulam, Hong Kong

## Abstract

There is no consensus guideline concerning the management of chronic hepatitis C patients during chemotherapy, and immunosuppression. However, there are some suggestions in literature that hepatitis C viral load increases during chemotherapy and there is a risk of rebound immunity against hepatitis C after discontinuation of immunosuppression with a consequent liver injury. A close monitoring of liver function of these patients is prudent during treatment of haematological malignancy. Antiviral treatment is deferred after the completion of chemotherapy and recovery of patients' immunity to minimize the toxicity of treatment. A combination of pegylated interferon and ribavirin is the standard therapy in hepatitis C infected haematological patients.

## 1. Introduction

It is well known that reactivation of hepatitis B is a potential lethal complication of chemotherapy and prophylactic antiviral drugs are prudent during treatment of haematological malignancies. However, on the contrary, it is not certain how chronic hepatitis C infection would affect the outcome of haematological malignancies and bone marrow transplantation patients. There are case reports of severe flare up of chronic hepatitis C in patients undergoing chemotherapy [1, 2] but case series review found such complications uncommon [3]. On the other hand, long-term survivors of bone marrow transplantation recipients are more prone to the complications of hepatitis C infection [4] and treatment in this group of patients seems warranted. However, there is scanty data on management of chronic hepatitis C in haematology patients.

Hepatitis C is associated with development of haematological diseases ranging from immune thrombocytopenia to lymphoma. The underlying pathogenesis and its impact on treatment are discussed.

## 2. Hepatitis C

There are six major genotypes of hepatitis C and more than 50 subtypes. Genotype 1b is the most common subtype worldwide. About 170 million people have chronic hepatitis and the estimated annual incidence of new case of hepatitis C is 3 to 4 millions [5]. In a study in Europe, the approximate prevalence of chronic hepatitis C among bone marrow transplantation recipients is 6% [6].

Sixty to eighty percent of patients develop chronic hepatitis C after acute infection. About twenty percent of these patients will be complicated by cirrhosis in twenty to thirty years, and some of them may develop hepatocellular carcinoma [5, 7]. It is believed that successful control of viral replication by effective antiviral treatment would prevent such complications in these patients [8, 9]. 

## 3. Status of Hepatitis C during Chemotherapy and Immunosuppression

The level of hepatitis C viral RNA in blood has been shown to increase during chemotherapy and immunosuppression. At the same time, for those with pre-existing liver dysfunction, the transaminase levels often normalize during immunosuppression. Upon the withdrawal of chemotherapy or immunosuppressants, the hepatitis C viral RNA decreases with a concomitant rise in transaminase levels. In a study of ten chronic hepatitis C patients, the alanine transaminase level decreased in eight patients while all of them demonstrated a rise in hepatitis C RNA during a seven-week course of prednisone whereas there was a rebound of alanine transaminase upon the withdrawal of steroid in seven of them [10]. None of these patients developed fulminant hepatitis. Similarly, in another study of lymphoma patients who received rituximab with combination chemotherapy, the hepatitis C RNA increased during chemotherapy and declined after completion of treatment. The use of rituximab in patients without hepatitis seldom leads to hepatotoxicity. However, in this cohort, one patient developed significant hepatotoxicity during rituximab chemotherapy [11].

This fluctuation in hepatitis C viral load and liver enzymes during and after chemotherapy or immunosuppression is explained by the suppression of immunity during chemotherapy and a rebound of reaction towards hepatitis C upon its withdrawal. 

However, despite the above observations, the clinical impact of chronic hepatitis C infection on patients undergoing bone marrow transplantation or chemotherapy is not well characterized. In a study in United States, among thirty three chronic hepatitis C patients, only eighteen of them (55%) developed mild to modest elevation of liver enzymes during chemotherapy [3]. None of them developed severe flare up that required cessation of chemotherapy. On the contrary, a study in Europe reported that up to 65% of the patients with chronic hepatitis C infection developed significant hepatotoxicity during chemotherapy [12]. This high incidence of hepatotoxicity led to interruptions in chemotherapy and jeopardized the clinical outcome of these patients. In another study of 132 patients, five patients had to discontinue chemotherapy because of severe hepatic dysfunction during treatment [13]. In a recently published review of 160 chronic hepatitis C patients with non-Hodgkin lymphoma, twenty-four (15%) of them developed significant liver toxicity during chemotherapy [14]. The overall survival of chronic hepatitis C patients is also shown to be significantly worse than those without hepaitits C infection. At a median followup of two years of patients diagnosed with diffuse large B cell lymphoma in Groupe d'Etude des Lymphomes de l'Adulte (GELA) programs, the overall survival was 56% among chronic hepatitis C patients versus 80% in those without (*P* = .02) [12].

The reported incidence of hepatotoxicity in chronic hepatitis C patients undergoing chemotherapy varies greatly among these studies. In addition, because of the bleeding tendency commonly seen in haematology patients, all these studies only monitored liver enzymes and hepatitis C RNA level but none of them was based on histological evidence. Therefore, there is yet concrete data on the effect of chronic hepatitis C infection in patients receiving chemotherapy and more studies are needed in this aspect.

## 4. Hepatitis C in Hematopoietic Stem Cell Transplantation

Hepatitis C infection is associated with an increased risk of veno-occlusive disease (VOD) and graft-versus-host disease (GVHD) of liver. The reported incidence of VOD was 14% among those with chronic hepatitis C infection while it was only 8% in transplant recipients who were negative for hepatitis C. The chronic inflammation in hepatitis C-infected liver causes endothelial changes in the hepatic sinusoids and this may predispose the patients to VOD during bone marrow transplant [15].

Although significant liver dysfunction during or immediately posttransplant is uncommon, bone marrow transplantation recipients with chronic hepatitis C infection have a significantly worse long-term outcome. The estimated incidence of cirrhosis at twenty years post bone marrow transplantation is 24% in those with chronic hepatitis C [16]. Moreover, there is evidence that the annual fibrosis progression rate is significantly higher in posttransplantation patients than those chronic hepatitis C patients without transplantation [4]. In fact, chronic hepatitis C infection ranked the third as a cause of late mortality, after infections and GVHD in posttransplant patients [16]. Once they developed cirrhosis, their survival outcomes are markedly compromised.

## 5. Treatment of Hepatitis C in Haematological Patients

The current standard of treatment of chronic hepatitis C infection is a combination of pegylated interferon and ribavirin. There is currently no specific guideline for treatment of the infection in haematological malignancy patients. In these patients, including bone marrow transplantation recipients, hepatitis C treatment is deferred until patients' immunity and bone marrow recover. In particular, for allogeneic stem cell transplantation patients, hepatitis C treatment should be withheld until all immunosuppressants are tailed off and GVHD is completely resolved. Interferon is known to suppress bone marrow hematopoiesis and this would aggravate the cytopenia frequently seen in postbone marrow transplantation patients. Moreover, it is reported that the use of interferon in allogeneic bone marrow transplantation patients might trigger GVHD, and its use in stem cell recipients should be cautious [17]. Ribavirin may suppress erythropoiesis and this may aggravate the degree of anemia in hematology patients. The use of erythropoietin however, is shown to reduce the transfusion requirement of these patients. 

Combination therapy of pegylated interferon and ribavirin is shown to produce a sustained virological response, that is, undetectable hepatitis C RNA six months off therapy, in 20% of chronic hepatitis C patients after bone marrow transplantation. This response rate is compared unfavourably with that reported for the general population. Moreover, 30% of the studied patients did not receive this treatment because of the presence of contraindications [18]. The poor tolerability among hematology patients and their inferior treatment outcome warrant more research in this particular group of patients.

## 6. Hepatitis C and Autoimmune Cytopenia

Chronic hepatitis C infection is associated with thrombocytopenia, and the cause is multifactorial in most patients. There are various possible explanations, which include increased sequestration and destruction of platelets in patients with hypersplenism, reduced thrombopoiesis as a result of decreased production of endogenous thrombopoietin by diseased liver, direct marrow suppression by hepatitis C virus as well as dysregulation of immunity leading to autoimmune thrombocytopenia. One study demonstrated the presence of increased level of antiplatelet glycoproteins in serum of chronic hepatitis C infected patients with thrombocytopenia [19]. A significant proportion of these patients responded to standard immune thrombocytopenia therapy such as steroids and intravenous immunoglobulin. In two of the studied patients, the platelet count normalized after receiving pegylated interferon plus ribavirin therapy. The rise of platelet count in one of these patients coincided with the disappearance of hepatitis C RNA in blood [19]. Although it is well known that interferon causes cytopenia, its administration in hepatitis C infected patients with cytopenia is not necessarily contraindicated but should be evaluated on an individual basis.

## 7. Hepatitis C in Lymphomagenesis

It is well known that hepatitis C is a lymphotropic virus and is able to infect mononuclear cells in peripheral blood. Chronic hepatitis C infection is associated with various lymphoproliferative disorders and the most commonly reported association is mixed essential cryoglobulinemia. More than 95% of patients with mixed cryoglobulinemia have evidence of exposure to hepatitis C virus [20, 21]. 

B-cell clonality and t(14;18) translocation are both prevalent in the peripheral blood mononuclear cells of hepatitis C infected patients [22]. It has been shown that chronic hepatitis C infection is significantly associated with the development of non-Hodgkin lymphoma. The reported odds ratio ranged from 2 to 4, with the risk being more evident in area with a higher prevalence of hepatitis C infection [23]. The most commonly reported subtypes of non-Hodgkin lymphoma in chronic hepatitis C infected patients are marginal zone lymphoma and lymphoplasmacytic lymphoma. There are also reports on an increased prevalence of high grade B-cell lymphoma in hepatitis C infected patients but majority of them were arising from an underlying low-grade lymphoma.

It is found that CD81 is a hepatitis C coreceptor and it is expressed in B lymphocytes [24]. The engagement of CD81, which is part of the CD81/CD19/CD21 membrane complex, activate B lymphocytes and subsequently lead to their proliferation. As a result, this chronic antigenic stimulation may predispose to the development of B-cell lymphoproliferative disorders [25]. On the other hand, there are suggestions that chronic hepatitis C infection induces point mutations in both immunoglobulin and nonimmunoglobulin genes of infected B lymphocytes [26, 27]. It is, however, still controversial whether hepatitis C virus has a direct oncogenic effect. More studies are warranted before a definitive conclusion can be made.

## 8. Role of Antiviral Treatment in Hepatitis C-Associated Lymphoproliferative Diseases

As hepatitis C plays an important role in lymphomagenesis, it is postulated that eradication of virus may produce a response in its haematological manifestation as well. In a review of eighteen hepatitis C infected patients with indolent B-cell lymphoma, an overall response rate of the lymphoma after antiviral treatment alone was 63% and 80%, respectively, in the group receiving interferon plus ribavirin and pegylated interferon plus ribavirin, respectively [28]. There was, however, persistent presence of cryoglobulin in majority of the cases despite the absence of detectable tumour. In another study of nine hepatitis C infected patients with splenic marginal zone lymphoma, seven of them had sustained virological response after antiviral treatment (which consisted of interferon as first line therapy with ribavirin added if unsatisfactory response). All seven patients had a concomitant haematological response with a decrease in splenic size and disappearance of villous lymphocytes from peripheral blood [28]. In the remaining two patients who had persistent detectable hepatitis C RNA in blood, there was no significant clinical haematological response. The immunoglobulin gene rearrangement observed at diagnosis in these patients was still detectable in patients achieving a complete clinical remission. Although antiviral treatment has a therapeutic role in the treatment of hepatitis C-associated lymphoproliferative diseases, most of the patients still have detectable residual diseases by molecular methods. Antiviral therapy alone is unable to achieve a complete remission of their haematological diseases in these patients.

## 9. Conclusion

There are different opinions on the risk of hepatic dysfunction during chemotherapy for haematological patients with chronic hepatitis C infection. No consensus guidelines concerning the management of these patients are currently available and more studies on this issue are warranted.

The role of hepatitis C infection in lymphomagenesis is intriguing and a better understanding may help our management of hepatitis C-associated lymphoproliferative diseases. Although antiviral treatment may not be able to eradiate hepatitis C-associated lymphoma, it should be considered as a treatment option for tumour control, especially for those who may not be able to tolerate cytotoxic chemotherapy.
